# Nanomaterials for the treatment of bacterial infection by photothermal/photodynamic synergism

**DOI:** 10.3389/fbioe.2023.1192960

**Published:** 2023-05-11

**Authors:** Zhaochen Yan, Danqiu Wang, Yun Gao

**Affiliations:** Nursing Teaching and Research Department, The Fourth Affiliated Hospital of China Medical University, Shenyang, China

**Keywords:** nanomaterials, photothermal, photodynamic, Synergistic therapy, bacterial infections

## Abstract

In the past few decades, great progress has been made in the field of nanomaterials against bacterial infection. However, with the widespread emergence of drug-resistant bacteria, people try their best to explore and develop new antibacterial strategies to fight bacteria without obtaining or increasing drug resistance. Recently, multi-mode synergistic therapy has been considered as an effective scheme for the treatment of bacterial infections, especially the combination of photothermal therapy (PTT) and photodynamic therapy (PDT) with controllable, non-invasive, small side effects and broad-spectrum antibacterial characteristics. It can not only improve the efficiency of antibiotics, but also do not promote antibiotic resistance. Therefore, multifunctional nanomaterials which combine the advantages of PTT and PDT are more and more used in the treatment of bacterial infections. However, there is still a lack of a comprehensive review of the synergistic effect of PTT and PDT in anti-infection. This review first focuses on the synthesis of synergistic photothermal/photodynamic nanomaterials and discusses the ways and challenges of photothermal/photodynamic synergism, as well as the future research direction of photothermal/photodynamic synergistic antibacterial nanomaterials.

## 1 Introduction

Bacterial infection is a very common disease in people’s daily life, which is a serious threat to human health (Wu et al., 2018; Liu et al., 2019; Wu et al., 2019). Antibiotics are the main clinical treatment for pathogenic bacterial infections. However, in recent decades, due to unwisdom or abuse of antibiotics, drug-sensitive bacteria continue to mutate, leading to the emergence and prevalence of bacterial drug resistance (Crofts et al., 2017; Gross et al., 2019; Scheres and Kuszewski, 2019; Shang et al., 2020). It is necessary to focus on the development of treatments that can quickly and effectively overcome pathogenic bacteria without producing drug resistance (Laxminarayan et al., 2013; Wang et al., 2019; Sun et al., 2019; Yuan et al., 2020).

With the development of nanomedicine, more and more nanomaterials and new antibacterial therapy are used in antibacterial therapy (Huang et al., 2009; Shan et al., 2019; Yang et al., 2019; Murugesan and Scheibel, 2020). For example, silver nanoparticles (Zawadzka et al., 2021), metal oxides (Shi et al., 2018; Xie et al., 2020), carbon-based materials (Xi et al., 2019), and metal-organic frameworks (MOF) (Liu et al., 2019; Tao et al., 2023) have broad-spectrum antibacterial activity and can be sterilized by physical or chemical methods. Emerging antimicrobial methods, including PTT, PDT, and chemodynamic therapy, have recently been identified as effective antimicrobial methods and have attracted great attention in anti-infective therapy (Wang et al., 2020; Wu et al., 2023). Among them, compared with other treatments, phototherapy is favored because of its controllable, non-invasive, few side effects and broad-spectrum antibacterial properties (Liu et al., 2017, Liu, Guo, Li, Xiao, Zhang, Bu, Jia, Zhe, Wang and interfaces 2019; Ma et al., 2020). PTT uses photothermal agent near infrared (NIR) light to convert into local high temperature, thus destroying the cell membrane and denaturing bacterial proteins, thus achieving bacterial death (Mao et al., 2017; Wang et al., 2019; Qing et al., 2019). PDT uses photosensitizers to absorb energy under laser irradiation and transfer it to molecular oxygen to produce cytotoxic reactive oxygen species (ROS): hydroxyl radical (OH), superoxide anion (O_2_^−^), hydrogen peroxide (H_2_O_2_), and singlet oxygen (^1^O_2_) (Xia et al., 2017; Sun et al., 2019; Wang et al., 2020b). ROS can oxidize and destroy biomolecules, such as lipids, proteins, and nucleic acids, thus inducing cell apoptosis (Tan et al., 2018).

However, bacterial infection is a very complex process, including initial bacterial adhesion, biofilm formation and infection (Li et al., 2017). Therefore, the use of a single antibacterial method may not be enough, so the nanomaterials combined with a variety of antibacterial methods were studied to enhance the antibacterial effect. For example, hyperthermia and long-term exposure to single-mode PTT therapy may lead to inflammation and thermal damage to nearby normal tissue (Rizwan et al., 2014; Richter and Kietzmann, 2016; Zhu et al., 2018; Gao et al., 2019). Using single-mode PDT therapy to kill bacteria requires a large amount of ROS; while excessive ROS can damage normal tissue by inducing inflammation and necrosis (Choi et al., 2009; Ma et al., 2022; Wu et al., 2022). In addition, the short life of ROS will limit the role of PDT. Integrating PTT and PDT on a single platform can bring the advantages of both high fever and ROS to the treatment of infected sites under light irradiation, overcoming the inherent limitations of a single PTT or PDT (Mao et al., 2018; Jiang et al., 2020; Wang et al., 2021). PTT/PDT synergistic therapy shows great potential in overcoming the shortcomings of individual therapies to achieve enhanced antibacterial properties (Zhang et al., 2022). Based on the fact that the complex interactions between host and bacteria during bacterial infection lead to specific microenvironments, including low pH, hypoxia, toxins, enzymes and so on, scientists have developed multi-functional synergistic nanomaterials with the responsiveness of bacterial infection microenvironment (Koo et al., 2017; Lv et al., 2020; Li et al., 2022).

**FIGURE 1:**
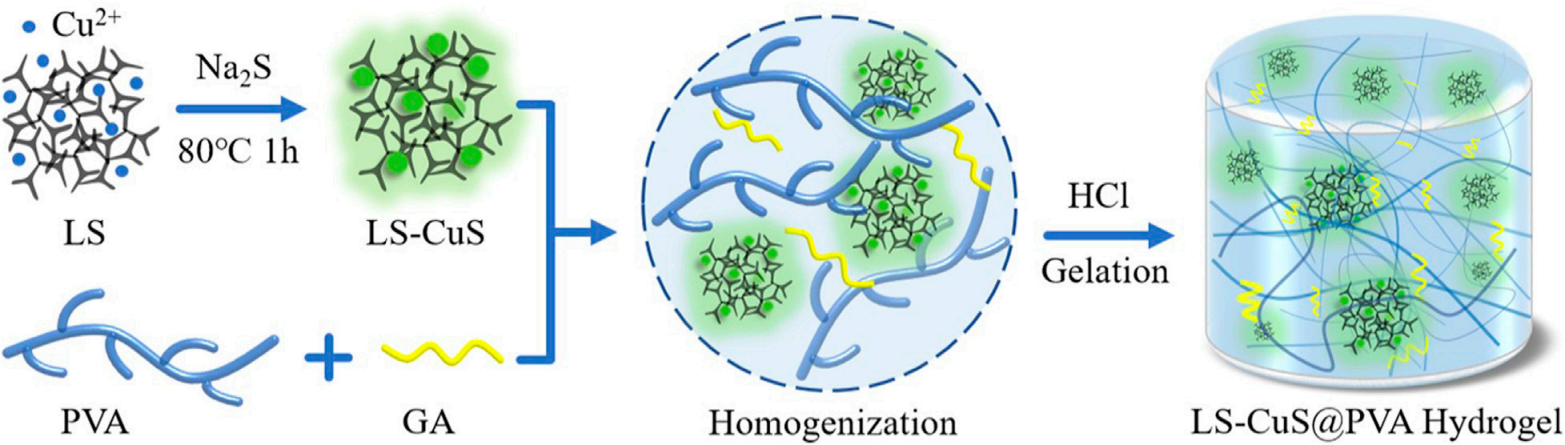
Photos of the LS-CuS@PVA hydrogel before and after gelation. Reproduced with permission from Ref (Xie et al., 2022).

This review introduces the latest progress of various nanomaterials used in the combination of PDT and PTT in the treatment of bacterial infections, such as hydrogels, multifunctional nanoplatforms, fiber membranes, nanosheets, and other nanomaterials, with emphasis on the loading and pathways of the materials driving the action of PDT and PTT, as shown in Figure 1. The recent overview of PDT and PTT strategies for the treatment of bacterial infections can provide clues to multiple ways of synergistic antimicrobial therapy and contribute to the development of new collaborative treatment systems to improve the efficacy of bacterial infection treatment, reduce side effects and avoid drug resistance.

## 2 Hydrogels for antibacterial therapy

Their porosity, biocompatibility, biodegradability, mechanical strength and stability are all adjustable, and provide excellent renewable 3D networks to simulate local tissue (Cheng et al., 2022). The porous structure of hydrogel is usually prepared by electrostatic interaction/hydrogen bond or covalent bond between the polymer chains, and their porosity, biocompatibility, biodegradability, mechanical strength and stability are all adjustable (Khurana et al., 2019; Sang et al., 2020; Maleki et al., 2021; Tao et al., 2022). It is suitable for the research and development of various materials (Liang et al., 2019a).

### 2.1 CuS -contained photothermal/photodynamic synergistic therapy

Copper sulfide (CuS) can show a significant photodynamic/photothermal effect under near-infrared light, produce hydroxyl radical (OH) in the presence of H_2_O_2_, and have excellent peroxidase-like activity. Because of its inherent advantages of near infrared absorption, efficient heat generation, low cost, biodegradability, and high cost, CuS has become an ideal material for antibacterial therapy (Wang et al., 2015; Wang et al., 2020c).

Recently, it has been reported that a LS-CuS@PVA composite hydrogel with near-infrared activated photothermal, photodynamic, and peroxidase-like activity was synthesized by introducing lignin copper sulfide (LS-CuS) nanocomposites into polyvinyl alcohol (PVA) hydrogels. The biodegradability of PVA polymer makes it a suitable scaffold for the construction of multi-functional antibacterial platform. The nano-gel can effectively kill bacteria through the synergistic antibacterial effect of photothermal, photodynamic and peroxidase-like activity, which is attributed to the local heat caused by photothermal effect, which destroys bacteria and makes them sensitive to ROS and accelerates the catalytic reaction to produce more OH *in vivo* and *in vitro*. It is proved that LS-CuS@PVA (Figure 1) has good efficacy in the treatment of antibiotic resistant bacteria and can inhibit the formation of biofilm (Xie et al., 2022).

Some CuS-treated hybrid hydrogels were synthesized. Trimethoxysilyl methacrylate (MPS, 97%) and mesoporous silica (MSiO_2_) modified CuS nanoparticles were synthesized by free radical polymerization. CuS nanoparticles can not only be used as photosensitizer of PTT, but also produce ROS in PDT under near infrared radiation. Under the irradiation of 808 nm near-infrared light, the near-infrared light of the mixed hydrogel is absorbed and converted into heat, then the copper ion formed by CuS NPs dissociation is released, and the OH produced by the reaction between the free carrier and water molecules under near-infrared light. The combined effects of high temperature, free radical oxygen species, and released copper ions under near infrared radiation make it have good antibacterial activity (Li et al., 2018).

The high surface activity of CuS nanoparticles makes it easy to agglomerate in the preparation process. Xiong et al. prepared uniformly dispersed CuS nanoparticles using corn straw as template and stabilizer, and then crosslinked with chitin to prepare CuS@cornstalk/chitin composite hydrogel. Under light, CuS nanoparticles embedded in hydrogel are released while producing light and heat, and hydrogen peroxide is decomposed to form strong oxidant OH, so as to realize the synergistic treatment of PDT and PTT (Xiong et al., 2019).

### 2.2 MoS_2_-contained photothermal/photodynamic synergistic therapy

Molybdenum sulfide nanosheets have their elemental abundance, electrochemical stability, high catalytic activity, and unique optical properties, and they have excellent photodetection capabilities in a variety of spectral responses, which can generate hyperthermia and ROS(Mak et al., 2010; Liang et al., 2018).

**FIGURE 2:**
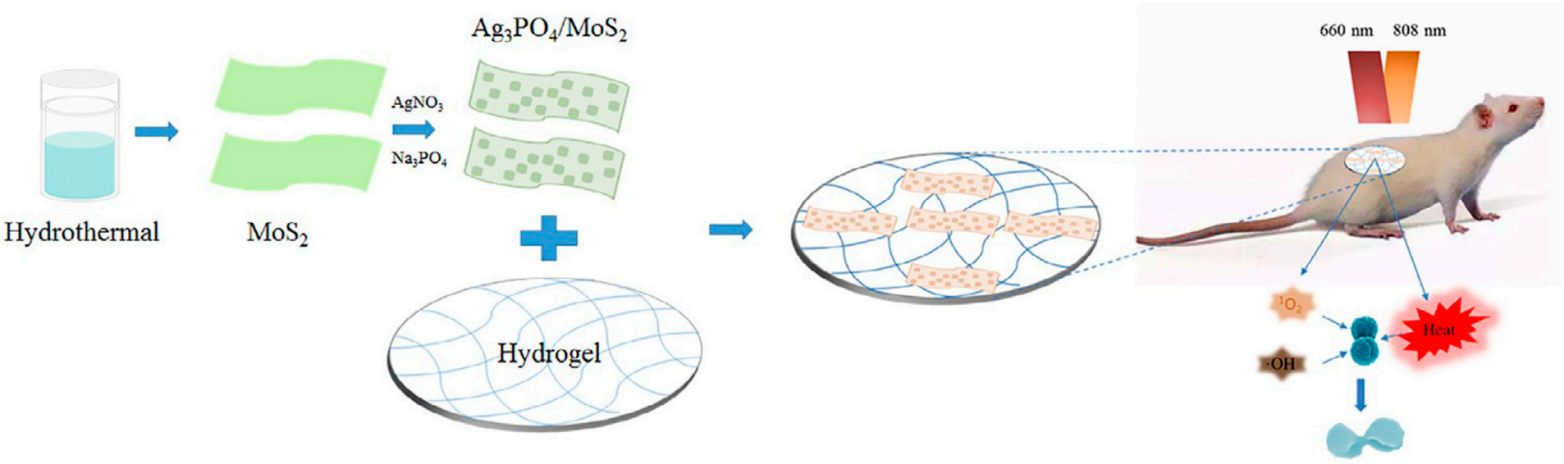
Schematic illustration of the synthesis of the Ag3PO4/MoS2 HD and its potential application in treatment of wound infection by the combination of PDT and PTT. Reproduced with permission from Ref (Zhang et al., 2019).

Zhang et al. prepared a composite hydrogel containing Ag_3_PO_4_ and MoS_2_. Ag_3_PO_4_/MoS_2_ composites were prepared by liquid phase reaction, and then dissolved in PVA to form the final product. Under 660 nm visible light (VL) irradiation, the hydrogel can be triggered to produce more ROS, while under 808 nm near infrared (NIR) irradiation, the hydrogel can produce more heat. Among them, Ag_3_PO_4_ can produce a large number of ROS, which can significantly improve the antibacterial activity and reduce the toxicity through the synergistic action of PDT and PTT, which also shows the advantage of synergistic antibacterial activity of PDT and PDT (Figure 2). However, it needs two kinds of light sources to achieve better results, which limits its application (Zhang et al., 2019).

A kind of hydrogel containing CuS and M_O_S_2_ is manufactured. The main step is to mix CuS@MoS_2_ microspheres into porous polyvinyl alcohol (PVA) hydrogels. The hybrid hydrogel reduces the excessive temperature of CuS@MoS_2_ microspheres under energy light irradiation to about 50 C, and the mixed hydrogel produces thermotherapy and ROS under double light (660 nm + 808 nm) irradiation. Based on the synergistic action of PDT and PTT, 99.3% of *Escherichia coli* and 99.5% of *Staphylococcus aureus* were killed in 15min due to the synergistic effect of photodynamic and photothermal antibacterial treatment under the irradiation of 660 nm VL and 808 NIR(Zhang et al., 2020).

### 2.3 ZnO-contained photothermal/photodynamic synergistic therapy

Zinc oxide not only has good biocompatibility and low cost but also has good performance in anti-inflammatory, antibacterial, antifungal, and other biomedical applications (Hahn et al., 2012; Sehmi et al., 2015; Surendra et al., 2016). Zinc oxide has the ability to produce ROS and can also be used to promote PDT therapy (Xiang et al., 2019; Xiang et al., 2020).

By introducing ZnO quantum dots@GO carbon nanotubes into the hydrogel structure, a zinc oxide quantum dots@GO nanocomposites (NCS) with good antibacterial activity was prepared. Graphene oxide (GO), as a new type of carbon material, has been widely used in the biomedical field because of its high light absorption in the NIR region (Gong et al., 2018; Zhang et al., 2018; Liang et al., 2019b). Under near-infrared light irradiation, GO in the nanocomposite can be used as a photosensitizer for PTT, while zinc ion can inhibit the action of respiratory enzymes and produce ROS, which irreversibly destroys bacterial cell membrane, mitochondria, and DNA, resulting in bacterial cell death, so as to achieve the combined effect of PDT and PTT (Liang et al., 2019a).

**FIGURE 3:**
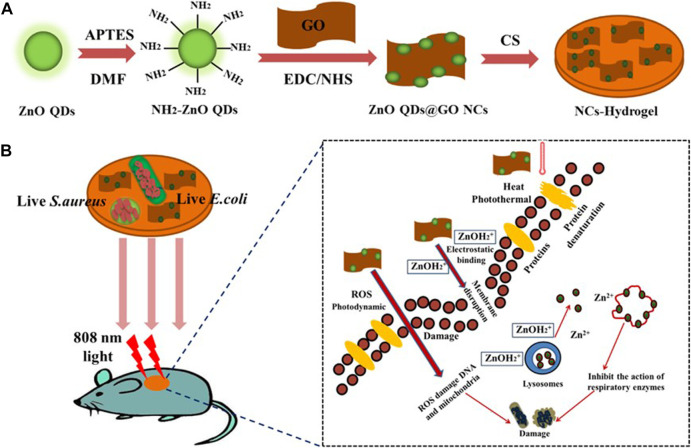
Schematic of the synthetic route of **(A)** ZnO QDs@GO-CS hydrogel and **(B)** bacteria killing processes. Reproduced with permission from Ref (Liang et al., 2019b).

Xiang et al. selected zinc ions in transition metal ions, and mixed hydrogels (DFT-hydrogel) were prepared with Folic acid (FA) and dopamine (DA). Firstly, carbon quantum dots (CQD) modified zinc oxide (C/ZnO) composites were selected as functional nanoparticles. PDA can be grafted onto its surface, which has good biocompatibility and excellent photothermal retention (Wu et al., 2018b; Chen et al., 2018; Liu et al., 2018; Nam et al., 2018). Two carboxyl groups in FA molecules or catechol in PDA can easily form metal-ligand coordination with zinc ions to form DFT-C/ZnO-hydrogel. Under the excitation of infrared or visible light, C/ethanol can produce reactive oxygen species (ROS), which can oxidize proteins, phospholipids and DNA/RNA in a very short time to achieve sterilization (Figure 3). In addition, CQD and PDA have good photothermal properties under near-infrared light, which is also helpful to sterilization. Therefore, the double irradiation of 808 nm near infrared light and 660 nm red light can enhance the treatment of PDT and PTT, and significantly improve the rapid antibacterial performance of the hydrogel (Xiang, Mao, Liu, Cui, Jing, Yang, Liang, Li, Zhu and Zheng, 2019).

### 2.4 Other photothermal/photodynamic synergistic therapy

**FIGURE 4:**
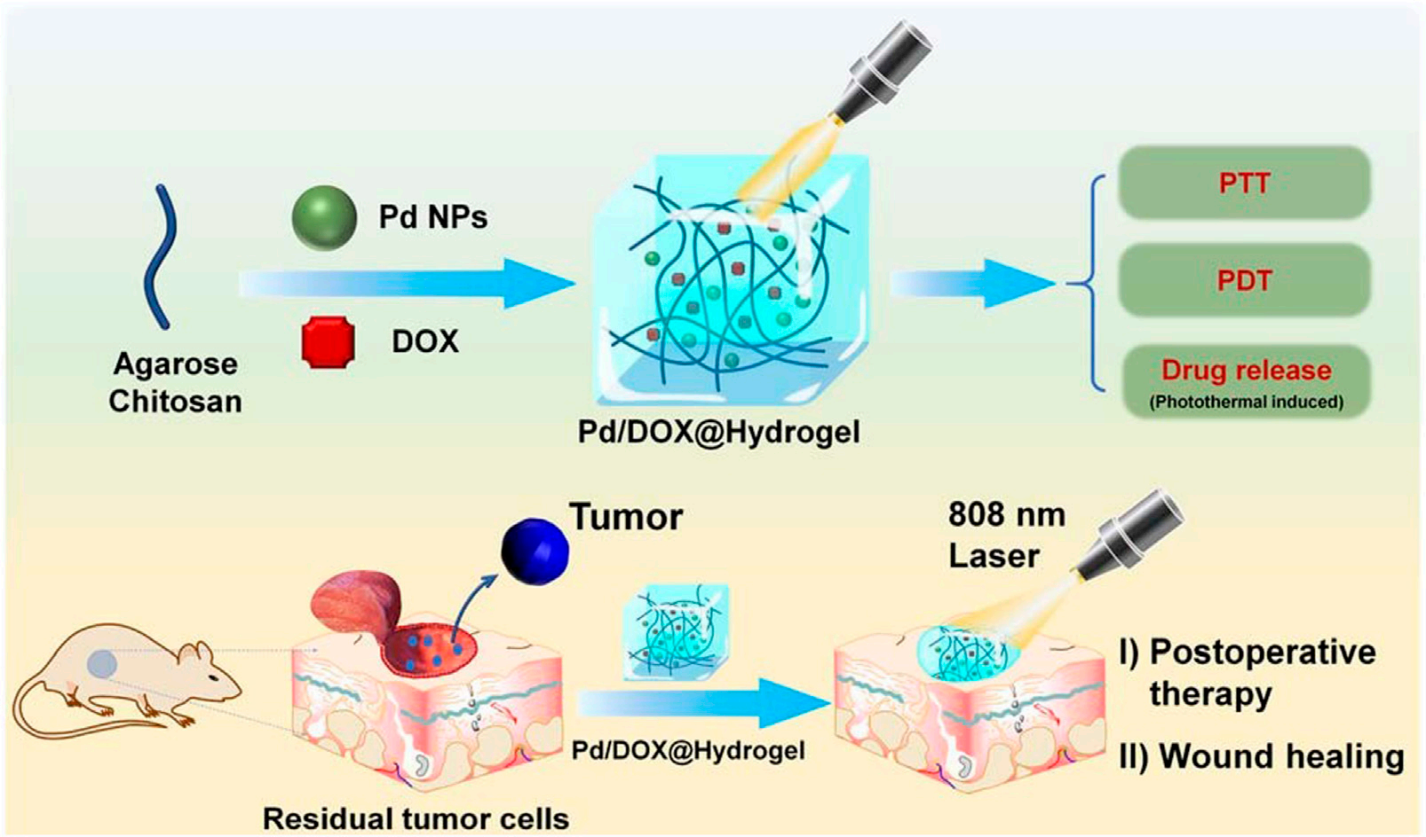
Schematic construction of Pd/DOX@hydrogel for post-operative therapy through NIR-light triggered photothermal/photo-dynamic therapy and drug release with wound healing capability. Reproduced with permission from Ref (Fan et al., 2023).

With the development of nanomedicine, the hydrogel can be used as a platform to explore the research and development of multifunctional therapeutic materials, not only in the treatment of bacterial infection but also in the treatment of tumors. For example, Yin et al. synthesized palladium nanoparticles (PdNPs) with PTT and PDT capabilities (Figure 4). Then the chemotherapeutic drug doxorubicin (DOX) was loaded on Pd nanoparticles to form hydrogel (Pd/DOX@hydrogel). Under the irradiation of near infrared light (808Nm), Pd/DOX@hydrogel produces enough heat to PTT, regulate drug release, and produce ROS, so as to further kill residual cancer cells and prevent wound infection (Fan et al., 2023).

## 3 Nanoplatforms for antibacterial therapy

Scientists have explored multifunctional materials based on PTT and PDT antibacterial models, such as metal materials carbon nanomaterials (carbon nanotubes, graphene, carbon dots, and nanocrystals), aggregation-induced luminescence materials (supramolecules based on metal rings) and halogen fluorescein (Zou et al., 2016; Xin et al., 2019; Wang et al., 2021; Niu et al., 2021). The antibacterial models of PTT and PDT show good application prospects in the field of sterilization and phototherapy (Han et al., 2020).

### 3.1 MoS_2_-contained photothermal/photodynamic synergistic therapy

**FIGURE 5:**
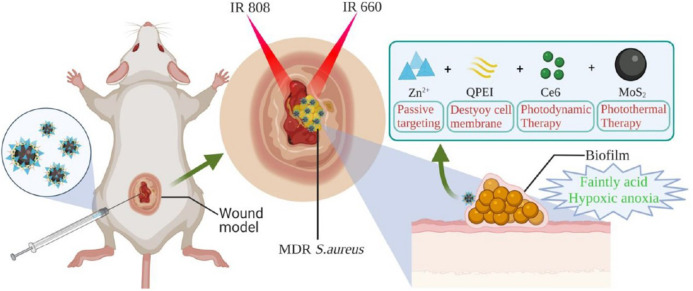
Schematic diagram of wound infection treatment using a combination of PTT/PDT/antibacterial active ingredients. Reproduced with permission from Ref (Jin et al., 2022).

Recently, Ge and his colleagues have developed a nanoplatform MoS_2_-QPEI/Ce6/PNS@ZIF-8 with dual response to pH and near-infrared light (NIR), which enables the synergistic antibacterial effect of PDT and PTT to promote wound healing. Firstly, quaternized polyethylenimine (QPEI) was added to the suspension of molybdenum disulfide (MoS_2_) nanosphere powder to form QPEI-Modified MoS_2_ Nanospheres and then mixed with dihydroporphyrin e6 (Ce6) and Panax notoginseng saponins (PNS), followed by the addition of zinc nitrate hexahydrate and 2-methylimidazole solution to form nano-platform MoS_2_-QPEI/Ce6/PNS@ZIF-8. The nanoplatform shows good dispersion and uniform nanometer size (120–150 nm) and has high photothermal conversion efficiency and good photodynamic effect. In the low acid microenvironment of the biofilm, the acid-sensitive zeolite imidazolium frame-8 (ZIF-8) decomposes and releases photosensitizer Ce6, MoS_2_; QPEI, and PNS. Photosensitizer MoS_2_ is used in photothermal therapy. The long positively charged carbon chain in the released QPEI structure adsorbs on the cell membrane surface through ion interaction and then destroys the cell membrane structure to release oxygen, alleviate the hypoxia state of the biofilm, and enhance Ce6-mediated PDT (produce^1^O_2_). PNS contains a variety of active ingredients to achieve antibacterial, hemostasis, and wound healing (Figure 5). *In vitro* antibacterial and live/death experiments showed that MQC@ZIF-8 achieved superior antibacterial activity through combined therapy (Jin et al., 2022).

**FIGURE 6:**
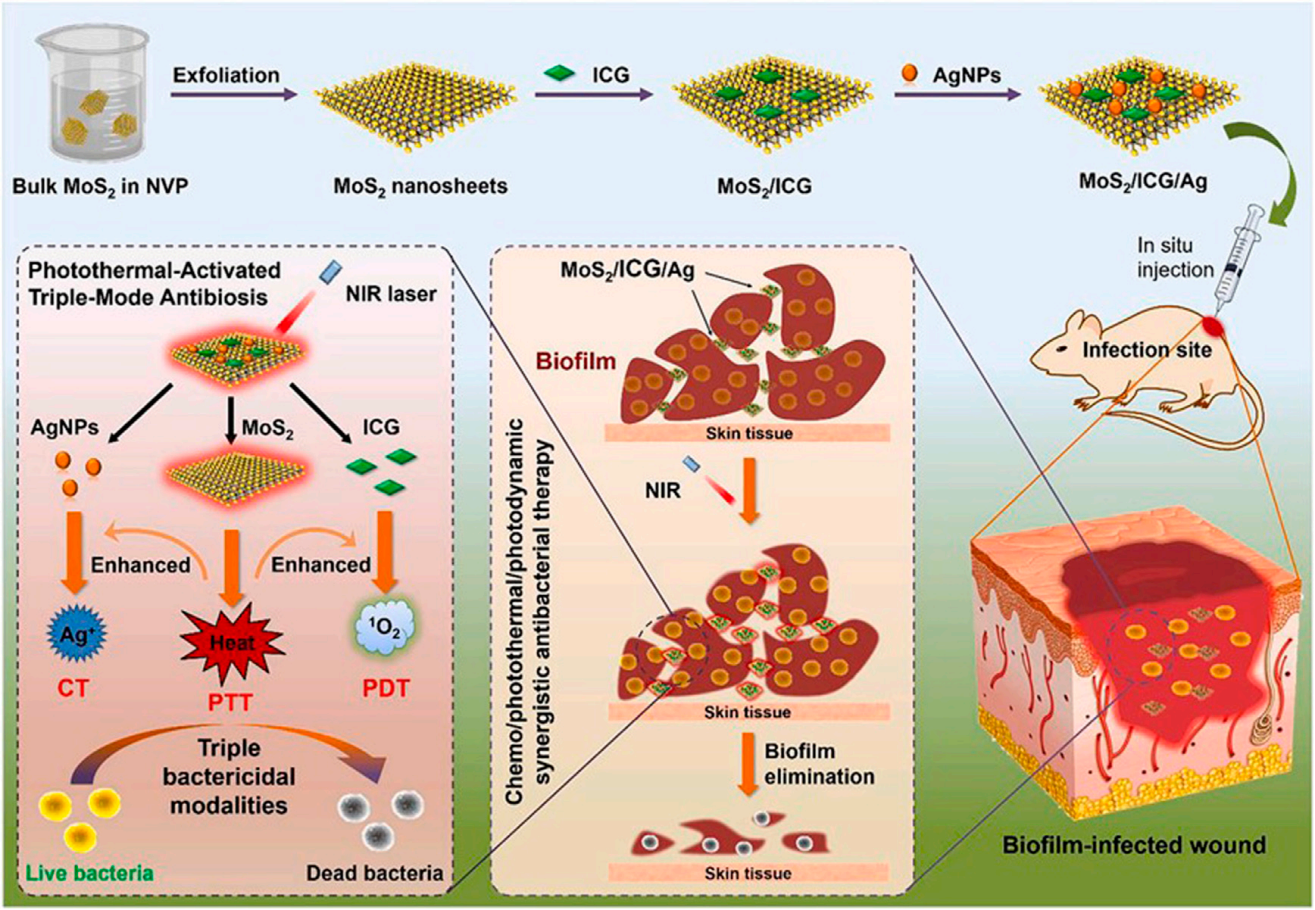
Schematic illustration for the preparation of multifunctional MoS2/ICG/Ag nanocomposites for the photothermally activated triple-mode synergistic antibacterial therapy. Reproduced with permission from Ref (Li et al., 2022).

A photothermally activated multifunctional nano-antibacterial platform was constructed by introducing indocyanine green (ICG) photosensitizer and silver nanoparticles (AgNPs) into the surface of molybdenum disulfide (MoS2) nanosheets. Photon hyperthermia produced by MoS2 nanoparticles can not only kill bacteria directly but also accelerate the release of ICG and silver ions, which are commonly used chemical antimicrobial agents. The released ICG can be converted into singlet oxygen with the help of photocatalytic oxygen of 808 nm, thus realizing photodynamic sterilization. And loaded ICG and AgNPs can in turn increase calories, which is a mutually reinforcing effect that can produce a synergistic therapeutic effect (Figure 6). The anti-infection experiment *in vivo* strongly proved that MoS2/ICG/Ag has significant anti-biofilm properties and low biological toxicity (Li et al., 2022).

Different Fe_3_O_4_@MoS_2_@sodium dodecyl sulfate nanocomposites were prepared on Fe_3_O_4_@MoS_2_ by ultrasound-assisted sodium dodecyl sulfate coating. The synergistic effect of Fe_3_O_4_@MoS_2_@sodium dodecyl sulfate and near infrared radiation eliminated almost all the biofilms of MRSA, thus improving the germicidal ability of Fe_3_O_4_@MoS_2_sodium dodecyl sulfate under near infrared radiation (Wang et al., 2022).

### 3.2 Cu-contained photothermal/photodynamic synergistic therapy

Copper ion not only has the ability to destroy bacterial membranes, but also has been proven to promote skin regeneration as a trace element.

A near-infrared (NIR)-activated chemical/photodynamic/photothermal composite therapeutic agent is loaded with fluorescein isothiocyanate (FITC) on mesoporous silica nanoparticles (MSN), super Small copper sulfide nanoparticles (Cu_2−_xSNPs) and polylysine (ε-Polylysine, PLL) were prepared. The biodegradable PLL can not only enhance the adhesion to the bacterial surface and increase the effect of phototherapy but also destroy the cells through electrostatic interaction. NIR-activated Cu_2_-xSNPs are used as popular PTT and PDT reagents due to their excellent photostability, negligible cytotoxicity, and biodegradability (Dai et al., 2021).

A hollow Cu_2-X_S nano-homojunction (nano-HJ) platform was developed by to effectively eradicate bacteria and tumors under tissue permeable near infrared (NIR) light. Copper ions released from Cu_2-X_Snano-HJs, Cu_2-X_S nano-HJS have the ability to destroy bacterial membranes, thus achieving the enhanced antibacterial effect in coordination with phototherapy. The nanocomposite material can not only detect bacteria and biofilm rapidly through fluorescence imaging but also ablate bacteria and biofilm through chemical/photothermal/photodynamic combined effects under near-infrared light irradiation (Gao et al., 2021).

**FIGURE 7:**
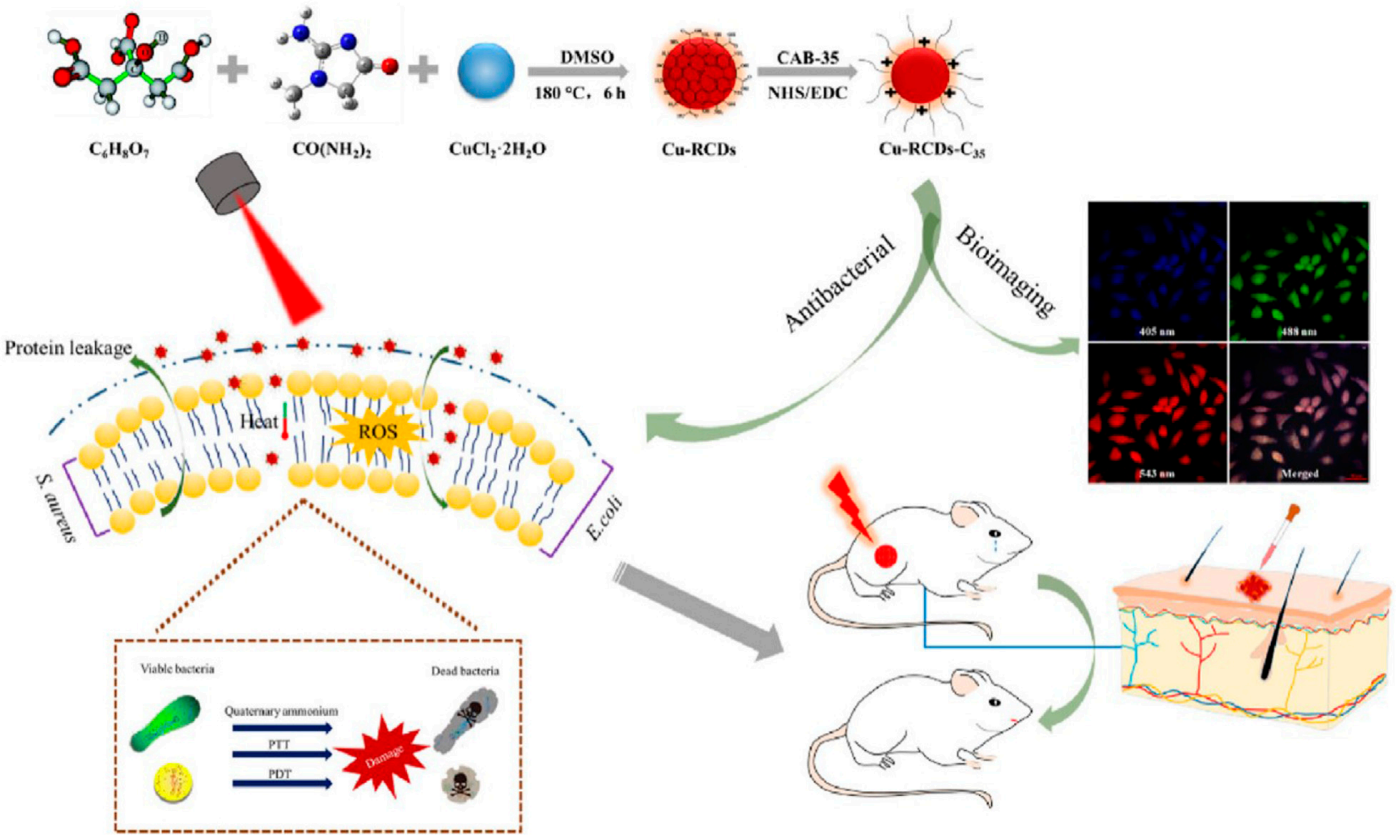
Schematic illustration of the preparation of Cu-RCDs-C35 and related biological applications. Reproduced with permission from Ref (Chu et al., 2021).

Chu and his colleagues successfully prepared a novel quaternary ammonium salted copper-RCDS by coupling the quaternary ammonium compound CAB-35 with copper RCDS through a simple preparation route. The quaternary ammonium group and long hydrocarbon chain in CAB-35 can destroy the cell membrane and enhance the sensitivity of bacterial cells to high temperatures and ROS. Under the irradiation of 808 nm laser, the synergistic antibacterial effect of PPT, PDT, and quaternary ammonium salt can be realized (Figure 7). The nanomaterials triggered by a single near infrared laser have PTT/PDT synergistic antibacterial properties, which can overcome the complexity of multiple light sources (Chu et al., 2021).

### 3.3 ICG-contained photothermal/photodynamic synergistic therapy

**FIGURE 8:**
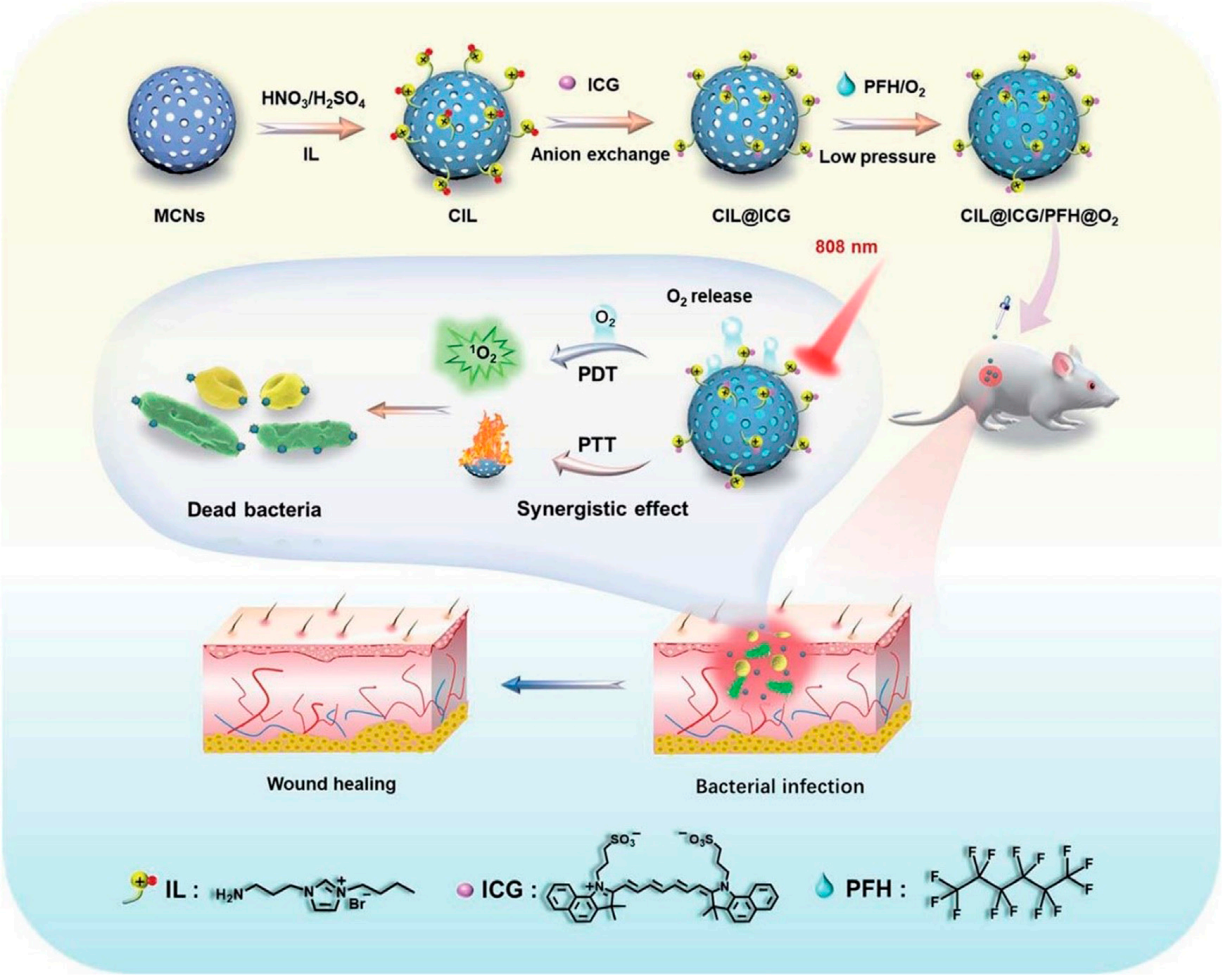
Schematic illustration of the synthesis of CIL@ICG/PFH@O2 nanoparticles and their corresponding synergistic antibacterial mech-anism under single wavelength (808 nm) NIR irradiation. CIL@ICG/PFH@O2 showed bactericidal activities against drug-resistant bacteria both *in vitro* and *in vivo* Reproduced with permission from Ref (Zhou et al., 2022).

The development of a mesoporous carbon nano-platform (CIL@ICG/PFH@O2) modified with cationic cations is reported. Cationic liquids attract anion ICG to provide a near-infrared triggered O_2_ diffusion enhanced PTT/PDT synergistic antibacterial therapy (Figure 8). Under the irradiation of single wavelength (808Nm) near infrared laser, carbon nanoparticles have a wide wavelength absorption range and high photothermal conversion efficiency, and their local temperature increases rapidly, which may promote the gasification of PFH and significantly accelerate the release of O2 from CIL@ICG/PFH@O_2_, thus rapidly activating and enhancing the photodynamic effect of CIL@ICG/PFH@O_2_ (Zhou et al. 2022).

### 3.4 Other photothermal/photodynamic synergistic therapy

Black phosphorus (BP) is found to have good photoluminescence properties, near-infrared photothermal absorption properties, which is the same as graphene (Li et al., 2014; Ling et al., 2015; Wu et al., 2018c; Zhao et al., 2018). Using this property, Liu and colleagues studied BP and AuNP nanocomposites (BPs@AuNPs). BPS has the ability to produce a large amount of ^1^O_2_ under the stimulation of 650 nm laser, which enables BPS@AuNPs to PTT/PDT under a single light source and has a synergistic therapeutic effect on bacteria. The nanocomposites have high antibacterial activity against *S. aureus* and *E. coli in vitro* and inhibit the growth of bacteria in the wound model of *S. aureus* infection *in vivo* (Liu et al., 2021).

Obeng et al. synthesized ZnO@Ag nanocomposites with good biocompatibility by doping ZnONPs with silver nanoparticles (AgNPs). Zinc oxide @ 8% Ag + PDT + PTT has a significantly destructive effect on biofilm. It has high antibacterial, antimicrobial membrane, and wound healing effects, and can be combined with PTT or PDT alone (Obeng et al., 2022).

## 4 Fiber membrane for antibacterial therapy

**FIGURE 9:**
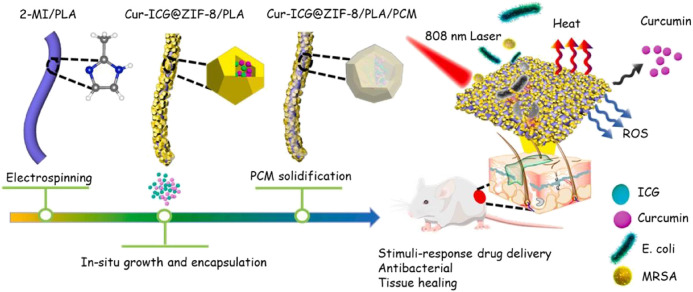
Schematic diagram of synthetic procedure and application of CIZPP. Reproduced with permission from Ref (Zhang et al., 2022).

An intelligent fiber membrane with multi-synergistic therapy has been developed to deal with drug-resistant bacterial infections. They first encapsulated curcumin and ICG in the large cavity of ZIF-8/PLA by chemical and electrostatic interaction and then coated the composite Cur-ICG@ZIF-8/PLA/PCM (CIZPP) with non-covalent interaction of phase change material (PCM). PCM has the mechanism of near-infrared induced phase transition and the dissociation of ZIF-8 in the acidic microenvironment of bacterial infection, which realizes the double stimulus response (NIR and pH) release of curcumin in CIZPP. In addition, due to the limiting effect, the photothermal stability and singlet oxygen (^1^O2) production of CIZPP were higher than those of ICG adsorbed directly on polylactic acid/PCM scaffolds. Indocyanine green (ICG) is an attractive PTT therapeutic agent to build a multi-functional treatment platform. The interaction between ICG and moderate ZIF-8/ICG strengthens the formation of ROS and promotes PDT (Figure 9). As an antibiotic-free antibacterial component, curcumin enhances chemotherapy. Through multi-synergistic anti-infective therapy, it can stimulate collagen deposition, promote the formation of the dermis and skin accessories, and effectively improve the healing rate of infected wounds (Zhang et al., 2022).

Zang et al. prepared titanium carbide (MXene)/imidazole framework-8 zeolite (ZIF-8)/PLA composite membranes by *in situ* growth of ZIF-8 molecular sieves on MXene and electrospinning. After MZ-8/PLA was compounded into electrospun scaffolds, it exhibited strong PTT and PDT properties under 808 nm laser irradiation. MZ-8 can promote the generation of ROS, and its photothermal conversion efficiency is 80.5%. *In vitro* experiments confirmed that the synthesized MZ-8 could generate hyperthermia and ROS based on the PTT-PDT effect, realizing the synergistic antibacterial effect of PDT and PTT (Zhang et al., 2021).

## 5 Nanosheets for antibacterial therapy

A new phototherapy nanoscale (MHCNSs) with the functions of hyaluronidase (HAase) response fluorescence imaging (FLI) and PTT/PDT was prepared. The Ce6 released by MHCNSs can produce ROS for antibacterial PDT, MoS_2_NSs and contribute to the bactericidal effect of MHCNSs, which makes MHCNSs a dual-mode (PTT/PDT) antibacterial nanoscale. The results of *in vitro* and *in vivo* experiments showed that MHCNSs had good biocompatibility. The study of antibacterial activity further showed that MHCNSS (40 μg mL^−1^) had an obvious killing effect on methicillin-resistant *S. aureus* in infected wounds of mice compared with other groups, and the reduction rate was 99.9% (Yuwen et al., 2021).

Recently, a new type of indocyanine green (ICG) functionalized hexagonal Mn_3_O_4_ nanoparticles (Mn_3_O_4_HNSs@ICG) has been designed to cooperate in the fight against bacterial infection. ICG as a photosensitizer, manganese oxide can convert light energy into high heat, and the released Mn^3+^ and Mn^2+^ ions participate in Mn_3_O_4_HNSs, which is conducive to the electron transfer in the Fenton-like reaction, thus promoting the production of ROS for treatment. Secondly, the flake structure with a rough surface and rich defects makes Mn_3_O_4_HNSs@ICG easy to adhere to the surface of bacteria, thus destroying its membrane system. Carry on the synergistic action of many ways to achieve good antibacterial. *In vitro* and *in vivo* toxicity evaluation showed that the material had good biological safety and was expected to be used in clinical anti-infective therapy (Zhang et al., 2022).

## 6 Other nanomaterials for antibacterial therapy

With the development of nanotechnology, more and more nanomaterials have been reported. There are other nanomaterials that can realize synergistic antibacterial effects of PDT and PTT have been developed.

Inspired by the morphology and infection mode of the COVID-19 coronavirus, Ni and his colleagues designed porous graphite nitride carbon (g-C3N4) with “artificial virus” embedded cobalt nanoparticles by self-assembling transpeptide transactivators with three layers of shell. Firstly, three layers of porous graphitized carbonitride (TCNCo) loaded with cobalt nanoparticles were prepared by the template method. The cobalt (Co) nanoparticles have an additional magnetic targeting function, which can enhance the ability of photothermal conversion. Then three-shell porous graphite carbon nanoparticles (TCNCo) coated with transduction peptide (TAT)were prepared by electrostatic self-assembly. TAT has a good ability to penetrate bacterial cell membranes because of its rich positively charged amino acids and stable secondary structure (Gao et al., 2019; Mookherjee et al., 2020). By imitating the coronal morphology and infection mode of COVID-19 cells, TCNCoT showed a tentacle-like structure on its surface, overcome the bottleneck of the bacterial membrane, successfully penetrated the bacterial cell membrane, and then released TCNCo with photothermal and photodynamic effects into the bacteria. *In vitro* experiments showed that the germicidal efficiency of the nanoparticles in 20min was as high as 99.99%, which was 18.6 times that of g-C3N4, and the germicidal efficacy remained 99.99% after 3 rounds of repeated use (Ni et al., 2022).

Self-assembled aggregation-induced emission (AIE) nanospheres (AIE-PEG_1000_ NPs) with near-infrared II (NIR-II) fluorescence emission, photothermal and photodynamic properties were prepared using multi-functional AIE luminescence (AIE-4COOH). AIE-PEG1000 nanoparticles were encapsulated in lipid nanoparticles with teicoplanin (Tei) and ammonium bicarbonate (AB) to form laser-activated nanoparticles (AIE-Tei@AB Nvs). (AIE) photothermal agent or AIE photosensitizer is one of the multifunctional materials with fluorescence imaging properties and PTT or PDT functions, so it can diagnose and treat diseases at the same time (Li et al., 2020; Zhang et al., 2021; Ni et al., 2021; Yan et al., 2021). Under the irradiation of 660 nm laser, AIE-Tei@AB NVS successfully realized the near infrared-II fluorescence and infrared (IR) thermal imaging of focus by loading the photoluminescence and photothermal properties of AIE-PEG_1000_ nanoparticles. At the same time, during the photothermal process, the loaded AB is thermally decomposed to produce a large number of CO_2_/NH_3_ bubbles (efficient ultrasound contrast agent), thus achieving high-performance ultrasound imaging of the infected focus The efficient photothermal and photodynamic characteristics of AIE-Tei@AB Nvs, combined with the decomposition and rapid release of NV during bubble formation. Synergistic treatment of bacterial infections through a variety of treatments (Li et al., 2023).

Using water as solvent and molybdenum trichloride (MoCl_3_) as the precursor, MoO_3_−xNDS nanozyme was prepared by a one-pot hydrothermal method. Based on the combination of photodynamic, photothermal, and peroxidase-like enzyme activities modulated by a single near-infrared irradiation (808 nm), MoO_3_−xNDS alone has a triple therapeutic synergistic efficiency. motivated by. Both photodynamic and nanozyme activities lead to the production of reactive oxygen species (ROS). The photothermal effect can adjust the MoO_3_−xNDS to their optimal enzymatic temperature (50 C), which can generate sufficient ROS even at a low concentration of H_2_O_2_ (100 µm). *In vitro* and *in vivo* experiments demonstrate the excellent antibacterial efficiency of MoO3-xNDs against drug-resistant extended-spectrum β-lactamases producing *E. coli* and methicillin-resistant *S. aureus* (MRSA) (Zhang et al., 2021).

## 6 Conclusions and prospects

This paper reviews the application of anti-infective materials containing PDT and PTT synergistic therapy in recent years. We discuss how various components in different materials play their roles in synergy. At the same time, we provide some latest examples that can overcome the shortcomings of a single treatment model by combining multiple approaches such as PDT and PTT, and achieve complementary multiple therapeutic effects. However, due to some challenges, the clinical application of these systems is still difficult to use. First of all, in order to improve the therapeutic effect and reduce the side effects, the combination of PTT and PDT has attracted much attention because of its low systemic toxicity, non-invasive, and excellent therapeutic effect. However, most PTT/PDT collaborative strategies are based on multi-component therapeutic agents prepared by complex processes and require different light sources to stimulate PTT and PDT. Therefore, it is very necessary to develop effective single-component drugs for PTT/PDT synergistic therapy. At the same time, simplification should be borne in mind when designing new synergistic therapeutic materials so that they can be used clinically by simplifying, expanding scale, and reducing costs. Secondly, the molecular mechanism of bacterial infection and the mechanism of drug resistance are not very clear. We need to evaluate the different characteristics of different bacteria in order to achieve accurate and efficient treatment. Then, the drugs that play the synergistic effect of PDT and PTT need to be released in an orderly manner and will not have harmful effects on normal cells, which requires an in-depth exploration of the synergistic therapy involved, which can make use of the differences between infected bacteria and normal cells for specific targeting. With the development of chemistry, material technology, and nanomedicine, the emergence of new design concepts and new treatments, as well as an in-depth understanding of the molecular and cellular mechanisms of bacterial infection, we believe that a new synergistic system that can meet the above challenges will be developed to further improve antimicrobial activity and reduce side effects and promote its clinical transformation in infection treatment.
